# Cryptococcosis in a seemingly immunocompetent patient presenting with knee and lower limb soft tissue infection complicated by meningitis: a case report

**DOI:** 10.3389/fmed.2026.1847042

**Published:** 2026-09-18

**Authors:** Zhongliang Huang, Junye Liang, Yibin Pan, Jinxiong Lao

**Affiliations:** 1 Foshan Hospital of Traditional Chinese Medicine, The Eighth Clinical Medical College of Guangzhou University of Chinese Medicine, Foshan, Guangdong, China; 2 Medical College of Acu-Moxi and Rehabilitation, Guangzhou University of Chinese Medicine, Guangzhou, Guangdong, China

**Keywords:** case report, Cryptococcus neoformans, high-throughput sequencing of pathogenic microbial nucleic acids, knee joint infection, soft tissue infection

## Abstract

Cryptococcus neoformans typically causes pulmonary or central nervous system (CNS) infections. However, osteoarticular manifestations, particularly as the initial presentation, are rare, especially in immunocompetent hosts. We report the case of a 65-year-old apparently immunocompetent woman admitted for generalized pain following a fall. Ten days post-admission, she developed fever and rapidly progressing soft tissue swelling in the right lower limb and knee. Despite the absence of overt immunodeficiency, the infection disseminated, leading to meningitis. Diagnosis was confirmed via fungal culture of lower limb secretions and metagenomic next-generation sequencing (mNGS) of knee joint tissue. Despite aggressive antifungal therapy, the patient's condition deteriorated, resulting in a fatal outcome. This case underscores the diagnostic challenge of cryptococcal osteoarticular infections in immunocompetent individuals. It highlights the utility of mNGS in diagnosing culture-negative infections and serves as a reminder that cryptococcosis should be considered in the differential diagnosis of fever of unknown origin with musculoskeletal involvement, even in the absence of traditional risk factors.

## Background

Cryptococcus is a subacute or chronic systemic mycosis caused by Cryptococcus neoformans, predominantly affects the central nervous system and lungs but can disseminate to virtually any organ (1). Cryptococcus neoformans commonly infects immunocompromised individuals, whereas Cryptococcus gattii occurs more frequently in immunocompetent hosts. The recognition of cryptococcal infections in non-HIV and seemingly immunocompetent patients is increasing due to a convergence of epidemiological shifts (Decline in HIV-Associated Cases, Rise of Other Immunocompromised States: hematologic disorders, diabetes mellitus, or tuberculosis etc.), an expanded understanding of subtle immune vulnerabilities (e.g., Genetic Susceptibility, Use of Immunomodulators), and the growing use of advanced diagnostics (e.g., mNGS, Serum Cryptococcal Antigen Test). This trend is not merely a matter of improved awareness but reflects real changes in the patient populations affected and the clinical scenarios in which the infection is identified.

While skeletal involvement occurs in 5%–10% of disseminated cases, isolated cryptococcal osteomyelitis is exceedingly rare (2). It typically affects immunocompromised individuals (e.g., HIV, organ transplant recipients) and primarily involves the vertebrae (3, 4).

Herein, we present a fatal case of disseminated cryptococcosis in a seemingly immunocompetent patient. The infection manifested initially as a knee joint and lower limb soft tissue infection, rapidly complicated by meningitis. This report aims to enhance clinical awareness of this atypical presentation and emphasize the diagnostic value of mNGS.

## Case presentation

A 65-year-old apparently immunocompetent female with a medical history of hypertension, osteoporosis, thoracolumbar compression fractures, and bilateral lower extremity venous thrombosis was admitted on November 18, 2025. The patient presented with a history of an accidental fall approximately two weeks prior to admission. The fall was immediately followed by the onset of localized pain involving the lumbodorsal region, bilateral gluteal areas, and the costal margins bilaterally. The pain was characterized as a persistent dull ache, with marked exacerbation upon positional changes—including transitions from supine to upright posture and turning in bed—as well as during weight-bearing activities, to the extent that independent ambulation was precluded. The patient remained fully conscious throughout the event, with no reported loss of consciousness or post-traumatic amnesia. Throughout the subsequent clinical course, no local signs of inflammation, such as erythema, warmth, joint effusion, or morning stiffness, were observed. Systemically, the patient reported no constitutional symptoms, including unintentional weight loss or night sweats (B-symptoms). Despite a trial of symptomatic therapy with oral analgesics, the pain failed to achieve meaningful relief and persisted, with a resting Visual Analog Scale (VAS) score of 7 out of 10. Before admission, the patient had no history of travel. Her family reported that pigeons had been cooked for consumption at home recently, but there was no definite history of patient's direct contact with the pigeons. Initial evaluation, including cranial CT and pelvic x-ray, revealed no acute abnormalities. The patient was afebrile and neurologically intact upon admission. Physical examination revealed no skin wounds or scraps.

After admission, laboratory findings were as follows: Complete blood count: WBC 10.87 × 10⁹/L, HGB 96 g/L, NEUT% 90.30%, NEUT# 9.82 × 10⁹/L, Lym# 0.61 × 10⁹/L. Coagulation function: APTT 22.1 s, D-Dimer 1.38 mg/L. Hepatitis B panel: HBsAg <0.05 IU/mL, Anti-HBs 683.50 mIU/L, HBeAg 0.16 COI, HBeAb 2.14 COI, HBcAb 3.06 COI. HIV-Ag/Ab 0.06 COI, Syphilis antibody 0.05 COI, HCV-RNA <500 IU/mL. Immunological assessment: C3 1.18 g/L, C4 0.450 g/L, IgA 1.89 g/L, IgG 7.23 g/L, IgM 0.65 g/L. During hospitalization, the patient received symptomatic treatments such as physical therapy and pain management. Due to the patient's advanced age and osteoporosis, recovery was slow. Although the patient's pain decreased, independent mobility remained difficult.

On November 28, 2025, the patient developed a fever (38.6 °C) and headache. By December 4, a subcutaneous nodule with inflammatory changes (suspected panniculitis) was observed above the right lateral malleolus (Figure 1A–E). Empirical antibiotic therapy (ceftriaxone and tigecycline) was initiated based on a positive urine culture for Escherichia coli.

**Figure 1:**
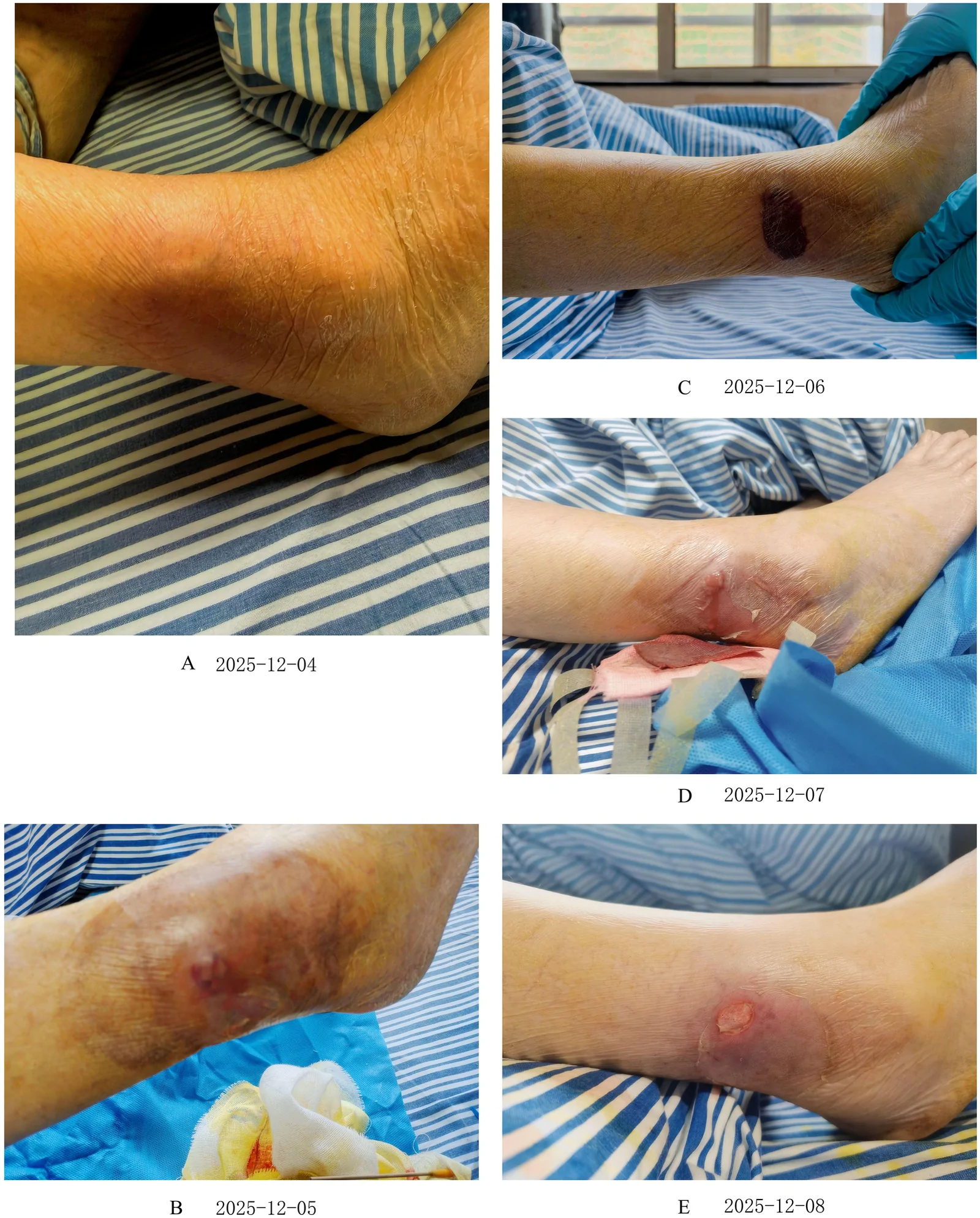
**(A–E)** Changes at the right ankle site infection site (December 4-8).

The clinical course rapidly deteriorated. On December 6, the right ankle lesion ulcerated with purulent discharge. December 8 imaging revealed acute cerebral infarction, soft tissue edema in the right lower leg, and effusion with synovial thickening in the right knee suggestive of septic arthritis. Synovial biopsy demonstrated granulomatous inflammation with necrosis (Figure 2).

**Figure 2:**
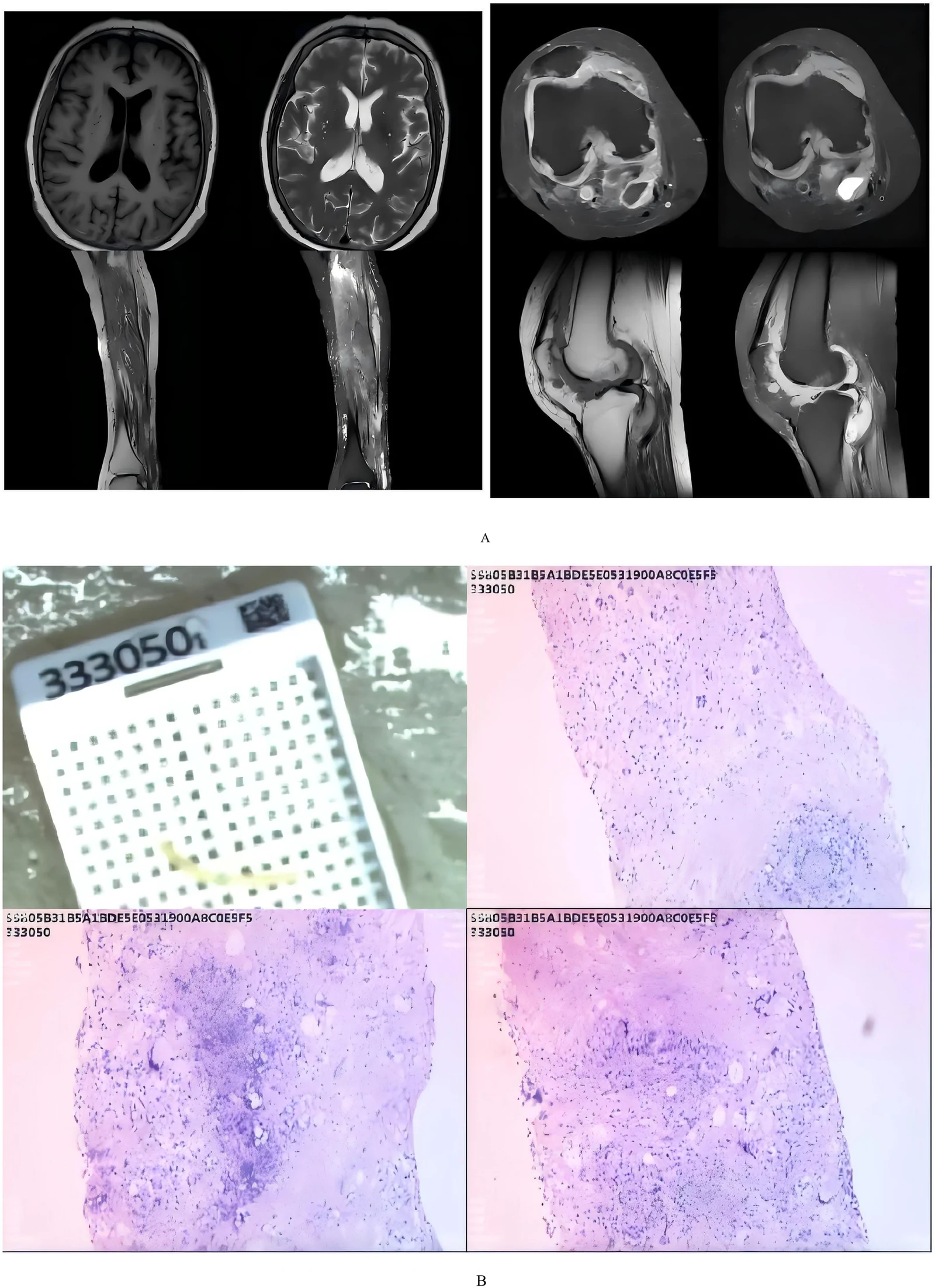
(A, B).

On December 10, etiological diagnosis was achieved: Aspiration of the right calf swelling yielded purulent discharge that grew Cryptococcus neoformans after 72 h of culture. Susceptibility testing revealed susceptibility to fluconazole (MIC 2 μg/mL), voriconazole (≤0.06 μg/mL), and flucytosine (≤4 μg/mL), but intrinsic resistance to echinocandins. Targeted metagenomic sequencing (MetaCAP™) of the formalin-fixed, paraffin-embedded (FFPE) tissue definitively confirmed Cryptococcus neoformans as the dominant pathogen, with 168,366 reads (99.96% fungal abundance, 13.93% genome coverage). The assay excluded bacterial, parasitic, and acquired resistance-gene co-infections, and detected only minimal EBV reads (*n* = 23), attributed to latent carriage. These molecular findings are entirely concordant with the positive culture and susceptibility results. Antifungal therapy with fluconazole was initiated immediately.

Despite treatment, the patient was transferred to a higher-level hospital on December 11 due to worsening neurological status. Cerebrospinal fluid (CSF) analysis confirmed cryptococcal meningoencephalitis (positive India ink stain, cryptococcal antigen titer 1:80, and positive CSF culture). Combination antifungal therapy (liposomal amphotericin B 0.2 g iv. qd, flucytosine 1.5 g P.O qid, and fluconazole 0.4 g iv. qd) was administered. In addition, Cefoperazone Sodium and Sulbactam Sodium for injection (2 g iv. q12h) was prescribed for antibacterial coverage. Unfortunately, the patient succumbed to the disease despite aggressive supportive care. The patient's rapid clinical deterioration and death precluded further diagnostic evaluation for potential underlying immunodeficiency disorders.

(Note: The tables provided in the original document are comprehensive. In a formal manuscript, ensure Table 1 is titled “Timeline of Clinical Events and Interventions”)

**Table 1:** Timeline of clinical events and interventions.

Date	Event/Symptoms	Findings	Interventions/Diagnoses
Hospital One 2025.11.18	Admission. Admitted due to “generalized pain for half a month after an accidental fall while walking.” Complex past medical history.	Alert. Pain in lower back, buttocks, and both knees; unable to walk.	Supportive care upon admission.
2025.11.19	Routine workup.	Complete blood count: elevated WBC, NEUT%; decreased HGB. Coagulation: elevated D-Dimer. Liver function: decreased albumin.	No specific intervention; continued observation.
2025.11.28	New symptoms. Developed fever (38.6 °C) and headache.	Unremarkable.	Antipyretic treatment administered.
2025.12.04	Disease progression. Swelling above the right lateral malleolus; persistent fever and headache.	Right lower limb ultrasound: suggested inflammatory changes (possible panniculitis). Chest CT: mild inflammation in both lungs. Urine culture(clean-catch midstream urine sample): Escherichia coli.	Antibiotic therapy with ceftriaxone and tigecycline initiated based on urine culture susceptibility results.
2025.12.06	Ulceration at the site of right ankle swelling with purulent discharge.	Discharge collected for testing. Influenza A + B antigen, procalcitonin, autoimmune antibody panel, etc., all showed no abnormalities.	Continued current antibiotic regimen.
2025.12.08	Acute deterioration. Recurrent fever, headache, slow reaction, swelling of right knee and ankle. Neurological scores indicated severe dysfunction (NIHSS 11).	Cranial MRI: acute cerebral infarction. Right lower leg MRI: hematoma formation; possible concurrent infection. Right knee MRI: proliferative synovitis suspected; possible septic arthritis. Right knee synovial biopsy: granulomatous inflammation with necrosis.	Antibiotic regimen adjusted to tigecycline + piperacillin/tazobactam.
2025.12.10	Etiological diagnosis. Metagenomic next-generation sequencing results from right lower leg discharge and right knee tissue returned.	Pathogen detection: Cryptococcus neoformans.	Tigecycline discontinued; antifungal therapy with fluconazole initiated.
Hospital Two 2025.12.11	Patient transferred to a higher-level hospital for continued treatment.	—	Patient admitted to Orthopedics; planned for lower limb/knee infection surgery. antifungal treatment to be continued.
2025.12.19	Diagnosis confirmed at higher-level hospital.	Cerebrospinal fluid (CSF) analysis: elevated opening pressure (320 mmH₂O), white blood cell count, and protein; decreased glucose and chloride. CSF culture + NGS: Cryptococcus neoformans, Epstein–Barr virus. Serum/CSF cryptococcal antigen: strongly positive (1:80 titer).	Definitive diagnoses: 1. Cryptococcal meningoencephalitis; 2. Disseminated cryptococcosis. Regimen adjusted to combination antifungal therapy with liposomal amphotericin B + flucytosine + fluconazole, along with antibacterial, anticoagulation, and supportive treatments.
Outcome	Patient died despite treatment.	—	—

[Note: The figures provided in the original document are comprehensive. In a formal manuscript, ensure Figure 1 is titled “Figure 1 A-E Changes at the Right Ankle Infection Site (December 4–8)”, Figure 2 is titled “Figure 2A” and “Figure 2B”]

## Discussion

Although cryptococcal infection is classically prevalent in HIV-infected populations, its incidence among immunocompetent individuals has been increasingly recognized in recent years. Diagnosing cryptococcosis remains a formidable challenge due to the paucity of specific clinical manifestations. This is particularly pronounced in immunocompetent hosts, where the rarity of the disease and atypical presentations predispose to diagnostic delay. In this case, the patient initially presented with isolated osteoarticular pain devoid of overt signs of infection or systemic toxicity. Furthermore, routine laboratory parameters failed to suggest a fungal etiology, rendering early diagnosis exceedingly difficult. Although Escherichia coli was isolated from urine cultures, suggestive of a concurrent urinary tract infection, this finding alone could not account for the subsequent emergence of fever, headache, soft tissue swelling, and neurological deterioration. This illustrates a prevalent clinical pitfall: the presence of a definitive bacterial pathogen often leads to premature diagnostic closure, resulting in the oversight of concurrent opportunistic pathogens, particularly fungi.

Our patient lacked a documented history of classic immunodeficiency diseases. Nevertheless, the presence of baseline lymphopenia on admission warrants careful reconsideration of their strictly “immunocompetent” status. Lymphopenia, even if mild, can serve as a subtle indicator of compromised cellular immunity, potentially predisposing patients to opportunistic infections such as disseminated cryptococcosis. In the context of rapid clinical deterioration where comprehensive immunological profiling (e.g., T-cell subset analysis) is unfeasible, such hematological abnormalities should not be overlooked. They may represent an underlying, albeit occult, immune defect. Furthermore, emerging evidence highlights the association between adult-onset disseminated cryptococcosis and anti-interferon-gamma autoantibodies, particularly in patients without HIV. Therefore, we propose that baseline lymphopenia be interpreted as a potential red flag for immune dysfunction. In similar future cases, screening for anti-interferon-gamma autoantibodies and other specific immune markers is strongly recommended to fully elucidate the host's immune status.

Cryptococcal osteomyelitis predominantly involves the spine; initial symptoms are typically pain, which may progress to localized erythema, swelling, and tenderness. Over time, this can culminate in recurrent purulent discharge and sinus tract formation. While CT and MRI usually reveal osteolytic destruction and medullary abscesses, these findings lack specificity. Although Papadakis (5) reported tibial involvement and Li (6) documented radial osteomyelitis—demonstrating that invasion is not confined to the spine—knee joint involvement remains exceptionally rare (7). The clinical and radiological features of cryptococcal osteomyelitis often mimic bone tumors and tuberculous spondylitis. Consequently, definitive diagnosis relies on cryptococcal antigen (CrAg) testing, fungal culture, and histopathological biopsy (8). In this patient, right knee synovial biopsy revealed “granulomatous inflammation with necrosis,” a finding that, while suggestive, is not pathognomonic for cryptococcosis. In contrast, metagenomic next-generation sequencing (mNGS), such as the MetaCAP assay utilized herein, offers a culture-independent approach. It enables rapid, sensitive detection of diverse pathogens (bacteria, viruses, fungi, and parasites) from clinical specimens (9). By circumventing the impact of prior antibiotic exposure on culture yield, mNGS ensures diagnostic accuracy (10), significantly enhances diagnostic efficiency, and facilitates the timely adjustment of therapeutic strategies. Recent literature highlights that mNGS offers high sensitivity and a rapid turnaround time, making it especially valuable for diagnosing central nervous system or disseminated cryptococcosis in patients with negative conventional microbiological cultures or atypical presentations (11, 12). While mNGS significantly enhances diagnostic yield, clinicians must still interpret the results in conjunction with clinical manifestations, as the detection of Cryptococcus DNA does not always distinguish between active infection and colonization. Future diagnostic algorithms should consider integrating mNGS as a complementary approach to traditional methods, particularly for seemingly immunocompetent patients with unexplained neurological deterioration.

In this instance, there was no evidence of primary cutaneous inoculation. Instead, the disease manifested initially as a musculoskeletal infection, which rapidly disseminated across the blood-brain barrier to involve the central nervous system (CNS). Although the family reported a history of pigeon exposure, direct contact could not be definitively established. The patient was a retired urban resident. A comprehensive environmental assessment revealed an unremarkable living environment devoid of pets. Notably, there was no evidence of pigeon guano or other avian droppings in or around the patient's residence. Furthermore, the patient had no documented history of direct or indirect exposure to soil, decaying wood, or wild animals. Nevertheless, environmental exposure remains a plausible risk factor. The proposed pathogenesis (13) suggests that Cryptococcus neoformans enters the host via the respiratory tract and is phagocytosed by alveolar macrophages. The organism can traverse phagocytes and epithelial cells to access the pulmonary interstitium, lymphatic system, and/or bloodstream. Alternatively, it may exit phagocytes via exocytosis, disseminating as free yeast. Subsequently, it can be transported by peripheral blood monocytes across the blood-brain barrier, establishing new foci within various organ systems.

Regarding the origin of pulmonary cryptococcosis in this case, two possibilities exist: hospital-acquired infection or reactivation of a latent focus. Cryptococcus neoformans is widely encountered in the environment, and most immunocompetent individuals harbour latent pulmonary granulomas after early-life exposure. Reactivation occurs when host immune surveillance declines. Our patient experienced several hospital-related stressors that may have impaired immunity: acute trauma from the fall, prolonged immobilisation, and broad-spectrum antibiotic therapy (ceftriaxone plus tigecycline), which can disrupt microbiota-dependent immune regulation. These factors, collectively, could have exhausted local antifungal defences and triggered reactivation of a pre-existing dormant focus. Nosocomial cryptococcal infection is extremely rare (14). Although previous reports of reactivation often involve corticosteroids or immunosuppressants, our patient did not receive such agents. Nevertheless, the combination of trauma, immobility, and antibiotics may serve as a sufficient alternative trigger. We therefore conclude that this case more likely represents reactivation of a latent infection acquired before admission, rather than a new hospital-acquired infection.

Acute cerebral infarction is a severe complication of cryptococcal meningitis, with reported prevalence ranging from 13% to 54% and carrying an odds ratio of 9.1 for morbidity and 2.6 for mortality. In the present case, the patient's acute deterioration with NIHSS 11 and MRI-proven infarction can be explained by three interlinked pathophysiological mechanisms. The primary mechanism is infectious vasculitis: C. neoformans preferentially colonises the basal subarachnoid space, inducing intense granulomatous inflammation that encases small penetrating arteries, particularly the lenticulostriate and thalamoperforating branches. Perivascular inflammation triggers vessel wall thickening, intimal proliferation, and secondary thrombosis, leading to lacunar infarcts in deep grey matter structures—with thrombosis from inflammation, rather than direct fungal invasion, being the dominant cause. Additional vascular injury may arise from direct endothelial toxicity, immune-complex deposition, and vascular strangulation by basal exudates. The second mechanism is severe intracranial hypertension; the opening pressure of 320 mmH_2_O markedly reduces cerebral perfusion pressure, compromising blood supply to watershed zones and deep subcortical areas, thereby exacerbating ischaemic risk. Third, the systemic inflammatory response to fungal dissemination induces a hypercoagulable state through elevated inflammatory markers and coagulation cascade activation, increasing thromboembolic risk. These mechanisms act synergistically: local vascular pathology combined with low perfusion and systemic hypercoagulability creates a prothrombotic environment predisposing to both small-vessel lacunes and, occasionally, larger territorial infarcts. Clinically, this case underscores that cryptococcal infarction can occur in apparently immunocompetent individuals, and that infarcts are typically multiple and bilateral. Their presence portends a worse prognosis, highlighting the need for prompt antifungal therapy alongside management of raised intracranial pressure and coagulopathy. While histopathological confirmation of each mechanism is lacking, the constellation of clinical, laboratory, and imaging findings strongly supports this multifactorial pathogenesis.

Therapeutic management of cryptococcosis hinges on the early initiation of adequate and prolonged antifungal therapy. Following diagnosis, the patient was transferred to a tertiary center and administered a combination regimen of liposomal amphotericin B, flucytosine, and fluconazole. This triad represents a current standard of care for severe cryptococcal infections, particularly those with CNS involvement (15). Amphotericin B is a first-line agent, though its use is limited by a high incidence of adverse effects; the liposomal formulation mitigates nephrotoxicity. Flucytosine, which inhibits fungal nucleic acid synthesis, exhibits synergy with amphotericin B. Fluconazole, valued for its excellent CNS penetration, is standard for consolidation and maintenance. Despite this aggressive antifungal strategy, the patient succumbed to the disease. This outcome suggests that cryptococcosis, particularly disseminated infection with CNS involvement, follows a fulminant course with a poor prognosis. This fatality may be multifactorial, attributable to diagnostic delay, a high fungal burden, multiple comorbidities (e.g., hypertension, osteoporosis, venous thrombosis), and potential adverse drug reactions.

## Conclusion

The epidemiological landscape of cryptococcosis has shifted significantly over recent decades. While HIV-infected individuals remain the archetypal high-risk group, emerging evidence indicates that severe and even fatal Cryptococcus infections are increasingly prevalent among immunocompetent hosts (16, 17). Indeed, epidemiological studies estimate that 10%–40% of non-HIV patients with cryptococcosis lack identifiable immunocompromising conditions (16). Notably, a recent multicenter cohort study demonstrated that immunocompetent individuals constituted over half (57.1%) of cryptococcosis cases (18). In this report, the patient exhibited no underlying immunosuppressive disorders; her infection was likely acquired through environmental exposure, corroborating this evolving trend. Nevertheless this fatal case of cryptococcal meningoencephalitis in a 65-year-old patient highlights the lethal impact of immunosenescence on infectious disease outcomes. Aging-driven immune deterioration, characterized by thymic atrophy, naive T-cell depletion, and dysregulated inflammation (inflammaging), impaired pathogen clearance and exacerbated tissue damage. As demonstrated by Liu et al. (19), age-related immune remodeling compromises adaptive immunity, impairing defense against opportunistic pathogens like Cryptococcus neoformans. The accumulation of senescent immune cells and chronic pro-inflammatory states created a permissive environment for uncontrolled fungal proliferation and neurological destruction. This case underscores the critical role of immunosenescence in host vulnerability to invasive fungal infections, emphasizing the need for targeted interventions to reverse immune deficits and improve outcomes in elderly patients. Future strategies may involve modulating aging-related immune pathways to enhance antifungal immunity in this high-risk population.

This case underscores the diagnostic challenges associated with osteoarticular cryptococcosis. Radiologically, cryptococcal osteomyelitis or arthritis often manifests as osteolytic destruction with soft tissue swelling, mimicking primary bone tumors, metastatic lesions, or skeletal tuberculosis (6, 16). Consequently, in seemingly immunocompetent patients presenting with fever of unknown origin, refractory multi-site soft tissue infections, or concomitant neurological symptoms, clinicians must include rare fungal pathogens—specifically Cryptococcus—in the differential diagnosis. A meticulous history focusing on environmental and animal exposure (e.g., avian droppings) is crucial. Furthermore, the timely application of etiological investigations, including cryptococcal antigen (CrAg) testing and mNGS, is imperative to circumvent diagnostic delays. Given the cryptococcal infection, a comprehensive immunological profiling was imperative to ascertain underlying immune deficiencies. However, the precipitous clinical deterioration precluded the completion of planned diagnostic evaluations, thereby hindering the elucidation of potential immunological etiologies.

Currently, standardized treatment guidelines for osteoarticular cryptococcosis are sparse. Per the Infectious Diseases Society of America (IDSA) guidelines, localized non-meningeal infections are typically managed with fluconazole (400 mg/day) for 6–12 months. Conversely, disseminated infections or those involving the CNS necessitate induction therapy with liposomal amphotericin B combined with flucytosine, followed by fluconazole consolidation. Prompt initiation of antifungal therapy, rigorous monitoring for adverse drug reactions, and aggressive management of comorbidities are essential to optimize outcomes. Enhanced multidisciplinary collaboration and awareness of atypical presentations are pivotal for improving diagnostic accuracy.

In summary, this report offers three critical insights for clinical practice:

Vigilance in Immunocompetent Hosts: The differential diagnosis of disseminated infection should extend beyond immunocompromised patients to include seemingly immunocompetent individuals, particularly when infections are refractory to conventional antibiotics.
Diagnostic Utility of mNGS: Advanced molecular techniques, such as mNGS, are invaluable for rapidly identifying rare pathogens and mitigating diagnostic odysseys.
Prognostic Implications: Despite standardized antifungal regimens, disseminated cryptococcosis with CNS involvement carries a grim prognosis, highlighting the non-negotiable importance of early recognition and intervention.

Future research should focus on elucidating the host susceptibility factors in immunocompetent populations and refining antifungal strategies to ameliorate outcomes in this emerging cohort.

## Data Availability

The original contributions presented in the study are included in the article/Supplementary Material, further inquiries can be directed to the corresponding author.
